# Harnessing Digital Phenotyping for Early Self-Detection of Psychological Distress

**DOI:** 10.3390/healthcare13162008

**Published:** 2025-08-15

**Authors:** Jana G. Zakai, Sultan A. Alharthi

**Affiliations:** Department of Software Engineering, College of Computer Science and Engineering, University of Jeddah, Jeddah 21959, Saudi Arabia; jzakai.stu@uj.edu.sa

**Keywords:** mHealth, phenotyping, psychological distress, mental health, anxiety, healthcare

## Abstract

Psychological distress remains a significant public health concern, particularly among youth. With the growing integration of mobile and wearable technologies into daily life, digital phenotyping has emerged as a promising approach for early self-detection and intervention in psychological distress. **Objectives:** The study aims to determine how behavioral and device-derived data can be used to identify early signs of emotional distress and to develop and evaluate a prototype system that enables users to self-detect these early warning signs, ultimately supporting early intervention and improved mental health outcomes. **Method:** To achieve this, this study involved a multi-phase, mixed-method approach, combining literature review, system design, and user evaluation. It started with a scoping review to guide system design, followed by the design and development of a prototype system (ESFY) and a mixed-method evaluation to assess its feasibility and utility in detecting early signs of psychological distress through digital phenotyping. **Results:** The results demonstrate the potential of digital phenotyping to support early self-detection for psychological distress while highlighting practical considerations for future deployment. **Conclusions:** The findings highlight the value of integrating active and passive data streams, prioritizing transparency and user empowerment, and designing adaptable systems that respond to the diverse needs and concerns of end users. The recommendations outlined in this study serve as a foundation for the continued development of scalable, trustworthy, and effective digital mental health solutions.

## 1. Introduction

Today, we are highly dependent on technology, especially smartphones, which have become an integral part of our daily lives. These mobile devices generate vast amounts of data every second through both active and passive user interactions, capturing a detailed digital footprint of individual behaviors and routines [1]. This unprecedented and continually accelerating level of data generation is transforming the landscape of health research and practice [1,2,3,4]. Simultaneously, our engagement with digital devices has intensified, especially during and after the COVID-19 pandemic. The time spent on digital platforms and social media has risen by 61.7%, contributing to increased psychological distress in various forms, including anxiety, depression, stress, and loneliness, particularly among both young and older adults [5,6]. Research has increasingly suggested a strong correlation between excessive smartphone use and mental health challenges. Notably, problematic cell phone usage has been identified as a significant predictor of depression in young adults [7], highlighting the complex interplay between digital behavior and psychological well-being.

Given the ubiquity of smartphones and the richness of behavioral data they provide, leveraging this information for mental health monitoring is a promising and rapidly growing field. Recent developments have demonstrated the utility of mobile data in detecting behavioral health issues such as mobile application addiction [8], video game addiction [9], and stress [10,11]. These studies reflect a broader shift toward the use of technology in mental health interventions, with passive data collection offering a non-invasive yet powerful means of capturing early warning signs of psychological distress. Despite this progress, mental health has significantly deteriorated in the wake of the pandemic, highlighting a deepening crisis that affects individuals across all age groups [6]. The rising prevalence of mental health issues has been well documented, with studies emphasizing the urgent need for scalable, timely, and effective mental health solutions [12,13,14]. Traditional approaches to mental health diagnosis, which often rely on self-reports or clinical assessments, may fall short in capturing early symptoms, particularly among populations that are less likely to seek help.

Early detection of psychological distress is crucial to mitigating its long-term effects and improving mental health outcomes [15]. In this context, our study explores the potential of digital phenotyping as a data-driven, scalable solution for the early identification and self-detection of psychological distress. Digital phenotyping refers to the “moment-by-moment quantification of the individual-level human phenotype in-situ using data from smartphones and other personal digital devices” [16]. This method represents a paradigm shift in how we assess mental health, offering a continuous, real-time view into an individual’s mental state that traditional assessment tools often lack [17,18,19].

By harnessing digital phenotyping, we aim to build a system capable of detecting psychological distress on a broader scale. Our goal is to utilize smartphone-derived behavioral data to increase the accessibility and timeliness of psychological distress detection. To achieve this, we followed a multi-phase research process: first, we conducted a scoping review to synthesize existing evidence on digital phenotyping and psychological distress; next, we translated these insights into the design and development of a prototype system (ESFY) tailored for youth; finally, we carried out a mixed-method user study to evaluate the system’s functionality, user experience, and real-world applicability. In doing so, we contribute to the growing body of work demonstrating how technology can bridge the gap between early symptoms and timely mental health support, thereby improving the overall quality and reach of mental healthcare.

## 2. Background

The onset of psychological disorders, particularly anxiety-related conditions, often begins early in life. The average age of onset for anxiety disorders is just 11 years, with approximately 75% of cases developing by the age of 21 [20]. According to the National Institute of Mental Health, 31.9% of adolescents are diagnosed with some form of anxiety disorder, with 8.3% experiencing severe mental impairment as a result [21]. Gender disparities are also significant, with prevalence rates of 38.0% for females compared to 26.1% for males. Alarmingly, these figures may not reflect the full extent of the issue, as many cases remain underreported or undiagnosed in primary care settings [12]. These statistics point to a growing mental health crisis among youth, highlighting the urgent need for innovative early detection tools and intervention strategies.

Recent technological advancements, particularly the widespread use of smartphones and wearable devices, offer new opportunities to address this crisis [6,22]. Digital phenotyping has emerged as a powerful and promising method for capturing real-time behavioral data that reflects mental health states [23]. Through the use of sensors embedded in everyday devices, digital phenotyping allows researchers and clinicians to passively and actively gather data related to users’ physical movement, sleep patterns, social behavior, and even emotional states. Common data sources include location tracking, accelerometer readings, and metadata related to communication and app usage. This data can be broadly categorized into active and passive forms [17]. Active data involves explicit user input, such as responses to self-assessment questionnaires or mood tracking entries. Passive data, on the other hand, is collected continuously without any required user engagement, such as screen time, call duration, or physical activity logged via device sensors [17,19]. This dual approach enables a comprehensive and less intrusive method for monitoring mental well-being over time.

The cost-effectiveness and scalability of digital phenotyping make it especially valuable for large-scale public health initiatives. It is particularly promising for under-resourced regions, including low-income countries and underserved populations that face barriers to traditional mental health services [24]. Systems based on digital phenotyping can identify early signs of psychological distress and notify users or caregivers, allowing timely interventions before symptoms escalate [25]. Jain et al. [25] argue that when digital phenotyping data is “gathered and analyzed appropriately,” it can reshape our understanding of illness by providing a more nuanced and dynamic picture of a patient’s condition.

Numerous studies have validated the predictive power of digital phenotyping. Bufano et al. [26] demonstrated its capacity to detect symptom exacerbation and potential relapses in patients with mental disorders, highlighting its potential for continuous mental health monitoring. Onnela et al. [27] emphasized its equivalence, if not superiority, to traditional mental status exams in assessing cognition, mood, thought processes, and motor activity.

**Figure 1:**
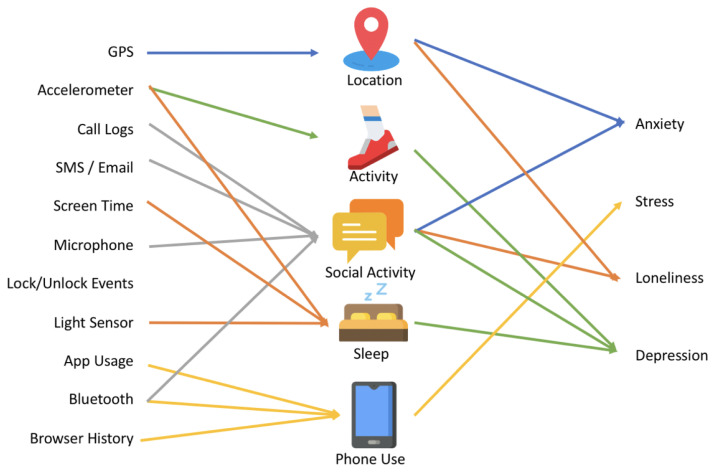
Relationships between digital phenotyping data, behavioral constructs, and psychological distress outcomes. Sensor inputs (e.g., GPS, accelerometer, screen time) inform behavioral indicators such as location, social activity, sleep, and phone use, which are associated with mental health outcomes, including anxiety, stress, loneliness, and depression. Adapted from Melcher et al. [1].

In addition to empirical studies, practical tools have also emerged. Torous et al. [28] developed LAMP, a smartphone-based clinical and research platform that operationalizes digital phenotyping in real-world mental health studies. Complementing this, Moshe et al. [29] provided a comprehensive review of digital interventions for depression, affirming the growing consensus that digital approaches are not only effective but essential for the future of mental health care. On the other hand, Melcher et al. [1] provided a foundational review of digital phenotyping for college student mental health, highlighting its relevance during the COVID-19 pandemic and beyond. Their work emphasized the feasibility of collecting both active and passive behavioral data through smartphones to inform mental health assessments. They highlight that common sensor-based streams, such as location, accelerometer, and screen usage, could be used to construct behavioral features associated with sleep, physical activity, and social interaction (Figure 1). These features are particularly significant as they correlate with mental health indicators like depression, anxiety, and stress. Melcher et al. [1] argued that college students represent a unique demographic well-suited to digital phenotyping due to their high smartphone adoption and willingness to engage with these technologies.

However, despite the promise of digital phenotyping, Melcher et al. [1] noted a lack of standardization across studies and limited efforts to scale these insights into deployable mental health tools. They proposed the development of more unified systems that could integrate behavioral signals and offer personalized psychiatric feedback or interventions. Our research builds directly on this recommendation. We adopted the data streams and behavioral markers identified in their review, such as sleep disruption, screen time, and reduced mobility, and embedded them into a coherent, prototype system (Figure 1). Our system design follows their call for real-world implementation by developing the ESFY, Expert System for Youth, an automated tool that uses smartphone-derived data to detect early signs of psychological distress in young users (Figures 3–5). By translating Melcher et al.’s [1] conceptual framework into a working prototype, we contribute to the operationalization of digital phenotyping for scalable mental health screening. In this work, we aim to develop an approach for mass early self-detection of psychological distress using digital phenotyping, building and expanding this prior work.

## 3. Methodology

This study adopts a user-centered design (UCD) [30] approach to develop a scalable solution for the early self-detection of psychological distress using digital phenotyping. Rather than beginning with a fixed system concept, our research is structured around understanding user needs and contextual insights drawn from prior literature. The study proceeds in three main phases: (1) a scoping review to synthesize current evidence and identify behavioral indicators and design considerations; (2) the development of a prototype system (ESFY) informed by those insights; and (3) a mixed-method evaluation to assess the system’s usability and functionality. This iterative, evidence-driven approach ensures that the system is grounded in the lived experiences, behaviors, and preferences of the intended users. Our study is structured in three main phases:

1. Scoping Review: We conducted a preliminary scoping review of recent literature on digital phenotyping and psychological distress. This exploratory approach helped us identify key behavioral markers and data streams, such as screen time, location, and social interaction, commonly associated with mental health outcomes. Insights from this review guided our subsequent system design.
2. Designing ESFY: Building on the insights from our review and Melcher et al.’s [1] conceptual framework, we developed ESFY: Expert System for Youth, a prototype system that collects and analyzes smartphone data to detect early signs of psychological distress.
3. Evaluating ESFY: We evaluated the ESFY through a mixed-method user study with participants. The study assessed usability, user experience, cognitive load, and the system’s ability to provide meaningful feedback. Feedback and results informed the system’s validation and future improvements.

The remainder of this paper is organized to reflect the sequential nature of our three-phase research process. First, we present the method for each phase: the scoping review, the design and development of the ESFY system, and the mixed-method user study. Next, we report the results of the scoping review and the user study. Finally, we present a comprehensive discussion that interprets the findings, addresses limitations, and outlines directions for future research on digital phenotyping in mental health.

### 3.1. Scoping Review Method

**Figure 2:**
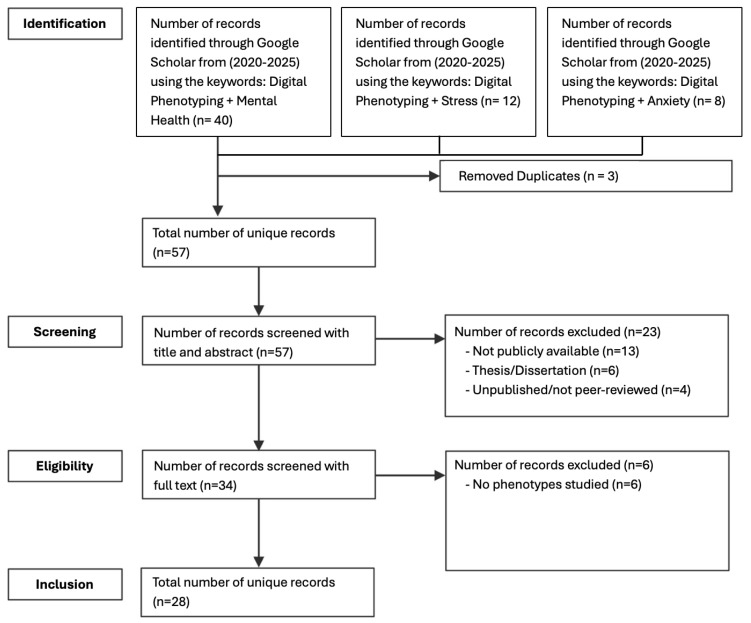
Flowchart of selection and inclusion process following the PRISMA statement.

In the first phase, we systematically explored and synthesized prior research to identify the most recent and relevant digital phenotypes and behavioral indicators associated with mental health outcomes (Figure 2). This scoping review was conducted in accordance with the PRISMA-ScR guidelines [31]. The review process involved clearly defining inclusion and exclusion criteria, systematically identifying relevant studies through targeted keyword searches in Google Scholar, and screening records at both the title/abstract and full-text levels by two independent reviewers. Discrepancies were resolved through discussion to reach consensus. Key data from the included studies were charted and synthesized using a structured thematic analysis approach [32] to map the current landscape of digital phenotyping research in relation to psychological distress. To ensure that the review captured the latest developments in this rapidly evolving field, we limited our search to papers published between 2020 and 2025. A set of targeted search strings were developed, including “digital phenotyping”, “mental health”, “anxiety”, and “stress”, to identify recent and relevant papers focused on the detection of psychological distress and the use of digital phenotyping.

Our literature search was conducted in Google Scholar, aligning with the search strategy employed by Melcher et al. [1], whose framework and findings substantially inform the present study. Google Scholar was chosen for its unparalleled breadth, indexing literature across a wide range of disciplines and publishers. While it is not typically recommended as the sole source for exhaustive systematic reviews [33], recent work has highlighted its particular value for initial scoping reviews in rapidly developing, multidisciplinary research areas [34]. This approach ensures broad and up-to-date coverage, which is essential for mapping recent advances in digital mental health research.

Moreover, several comparative evaluations have shown that Google Scholar often achieves higher recall than discipline-specific databases such as PubMed or PsycINFO, particularly when capturing gray literature, non-journal outputs, or recent publications that may not yet be indexed elsewhere [35]. Given the interdisciplinary nature of our study, Google Scholar offers an inclusive search environment that supports the identification of diverse and emerging contributions across domains. As such, we view our approach not as a limitation, but as a strategic methodological choice aligned with the scoping objectives of this review [34,35].

After collecting all relevant studies, their key findings, digital phenotyping mentioned, and type of psychological distress, were extracted and organized for analysis (Table 1). ATLAS.ti, a qualitative data analysis software application, was used to facilitate the systematic organization, coding, and synthesis of the review data. This process resulted in a total of 28 recent research articles that were included in the final data pool for analysis after removing all unrelated studies and duplicates.

Data from the scoping review were analyzed to systematically map the landscape of digital phenotyping research as it relates to the early detection of psychological distress. The primary aim was to identify the digital phenotyping modalities most commonly used, the types of psychological distress investigated, and the methodological trends and gaps present in the current literature. A thematic analysis approach [32] was employed, enabling the identification of recurring themes, associations between phenotypes and psychological distress types (e.g., anxiety, stress, depression), and emergent patterns across the literature. This rigorous and iterative process ensured consistency and depth of analysis, in alignment with prior studies (e.g., [6,36]).

Two independent researchers conducted the screening of studies at both the title/abstract and full-text levels. Studies were eligible for inclusion if they utilized digital phenotyping methods to assess or predict psychological distress, including but not limited to depression, anxiety, stress, or related constructs. Articles were excluded if they did not apply digital phenotyping or did not address psychological distress as a primary focus. In cases where disagreements arose regarding the inclusion or exclusion of specific studies, the researchers engaged in discussion to reach a consensus. The analysis followed a structured multi-step process.

- First, we familiarized ourselves with the dataset by thoroughly reading and reviewing the full text and extracted details of each included study, focusing on their use of digital phenotyping modalities and targeted psychological distress types.
- Next, we generated initial codes by systematically categorizing meaningful data excerpts, capturing recurring topics such as sensor type, data stream, mental health outcome, (e.g., anxiety, stress), methodological approach, design recommendations.
- After coding, we identified preliminary themes by grouping related codes into broader categories, such as mobility tracking for depression, social interaction data for loneliness, and multi-sensor integration for stress detection.
- These themes were then reviewed and refined to ensure that they accurately represented the underlying studies and were clearly distinct from one another. Examples of emergent themes included behavioral markers of anxiety, scalability of passive sensing, and gaps in loneliness detection.
- Finally, the refined themes were analyzed in depth and synthesized into a coherent narrative, highlighting the key findings, methodological strengths, and gaps in the digital phenotyping literature for psychological distress. This structured approach ensured that the review provided a comprehensive, evidence-based map of the field while also identifying directions for future research and system development.

With the final pool of studies systematically identified, screened, and analyzed as described above, we proceeded to synthesize the key findings to map current trends, identify dominant digital phenotyping modalities, and highlight common psychological distress outcomes addressed across the literature (Table 1). This synthesis informed both the conceptual foundation and functional requirements of the system developed in the next phase of our study.

**Table 1:** Studies included in the scoping review.

Paper	Study Type	Phenotypes Studied	Main Contribution
Hamilton et al. [37]	Empirical study	App usage, screen activity, accelerometer, communication	Tested the feasibility and acceptability of using smartphone-based mobile sensing (via AWARE app) to objectively track social media use among adolescents and proposed methods to improve digital phenotyping accuracy for mental health insights. In this study, they focused on depression, mood, and emotions.
Fransson et al. [38]	Empirical study	GPS, app usage, self-reported mental health surveys, voice diaries	This study combined passive smartphone data (GPS/mobility) and active self-reports from pregnant women during COVID-19 to track mental health changes. It found that reduced mobility and increased internet searches were linked to worse mental health, highlighting digital phenotyping’s value for real-time monitoring during crises. In this study, they focused on depression and anxiety.
Williams et al. [39]	Review	GPS, accelerometer, app usage, keystroke patterns, voice, camera	Digital phenotyping for mental health should be understood in relation to its environmental, spatial, and technological contexts, highlighting the need to consider how sensors, data, and environments interact. In this review study, they focused on mental health in general.
Choi et al. [40]	Review	GPS, microphone, accelerometer, call logs, Bluetooth, Wi-Fi, keyboard, SMS, emails, app usage, Gyroscope	This study explored the potential of smartphone sensors to detect behavioral patterns linked to stress, anxiety, and mild depression among nonclinical populations. The findings from the reviewed studies support the effectiveness of smartphone sensors in recognizing behaviors associated with these psychological conditions. In this review study, they focused on stress, anxiety, and mild depression.
Jacobson et al. [41]	Empirical study	Accelerometer, call logs, SMS	Demonstrated that smartphone sensor data can predict social anxiety severity using machine learning, with strong discriminant validity. In this study, they focused on social anxiety.
Jacobson et al. [42]	Empirical study	Wearable accelerometer	Demonstrated that wearable movement data can accurately predict GAD symptom severity using machine learning, with strong specificity and symptom associations. In this study, they focused on generalized anxiety disorder.
Cohen et al. [3]	Empirical study	Geolocation, accelerometer, screen state, active survey responses	Demonstrated that combined active and passive smartphone data can predict significant mood and anxiety symptom changes across users, supporting the feasibility of scalable symptom monitoring. In this study, they focused on mood and anxiety.
Zhang et al. [4]	Empirical study	physical activity, heart rate, sleep patterns	Used wearable data and machine learning to identify behavioral markers of depression and anxiety, showing the feasibility of large-scale mental health screening using digital phenotyping. In this study, they focused on depression and anxiety.
Nguyen et al. [43]	Empirical study	Pseudo-passive data, GPS, accelerometer	Applied machine learning to classify anxiety severity using survey-derived features during COVID-19 and introduced pseudo-passive features as a proxy for digital phenotyping. In this study, they focused on anxiety.
Kang et al. [44]	Study protocol	App usage, smartphone activity logs, wearable data	Introduced a hybrid research model combining centralized (clinical + app data) and decentralized (app-only) data collection methods to gather digital phenotyping data for mood and anxiety assessment at scale. In this study, they focused on mood and anxiety.
Egger et al. [45]	Empirical study	GPS, accelerometer, screen state, call logs, app usage	Proposed a framework to assess real-time stress and stress responses using passive smartphone data and digital phenotyping methods to enhance detection and early intervention in mental health. In this study, they focused on stress.
Shvetcov et al. [46]	Empirical study	GPS, accelerometer, app usage	Developed and validated a machine learning pipeline to predict stress levels in university students using passive smartphone sensing data. In this study, they focused on stress.
Melcher et al. [1]	Review	GPS, Accelerometer, social interaction data, app usage	Reviewed 25 digital phenotyping studies involving college students; highlighted the use of mobile sensing to monitor behaviors like sleep, social interaction, and exercise and emphasized the potential of these data streams for remote mental health assessment and personalized care. In this review study, they focused on mental health in general.
Mendes et al. [23]	Review	GPS, Accelerometer, Light sensor, Ambient data, App usage, Screen activity	Reviewed 31 sensing apps and 8 public datasets for digital phenotyping in mental health; highlighted key sensing modalities, gaps in dataset availability, and challenges in translating digital biomarkers into clinically actionable tools. In this review study, they focused on mental health in general.
Oudin et al. [47]	Perspective	GPS, smartphone interactions, and behavioral patterns	Discusses the conceptual and ethical implications of digital phenotyping in psychiatry, advocating for its integration into patient-centered care while cautioning against the depersonalization of therapeutic relationships. In this study, they focused on mental health in general.
Currey et al. [48]	Empirical study	GPS, call logs, sleep duration, screen state	Evaluated correlations between passive smartphone data and mental health survey scores in a large sample using the mindLAMP app. Found weaker correlations than smaller studies and highlighted the improved predictive value when passive data is combined with daily self-reports. In this study, they focused on stress, generalized anxiety disorder, loneliness, sleep quality, psychosis, depression and anxiety.
Currey et al. [49]	Empirical study	GPS, screen time, phone unlocks, app usage, sleep estimates	Validated a digital phenotyping model for predicting symptom improvement and guiding personalized interventions among college students, demonstrating its feasibility and engagement potential. In this study, they focused on stress, generalized anxiety disorder, loneliness, sleep quality, psychosis, depression and anxiety.
Birk et al. [50]	Review	Passive data, wearables, social media activity, behavioral tracking, sensor data	The paper critically examines digital phenotyping in mental health, highlighting ethical and conceptual challenges such as algorithmic bias, reductionism, and the need for greater reflexivity and social science involvement. In this study, they focused on mental health in general.
Adam, David [51]	Perspective	GPS, call logs, messaging frequency, battery usage, sleep patterns	Provides a synthesis of current efforts and challenges in using smartphone-derived digital phenotyping for mental health care, including its potential to predict relapse and symptom changes, while highlighting issues of privacy, platform fragmentation, and lack of large-scale validation. In this study, they focused on mental health in general.
Langholm et al. [52]	Empirical study	Phone usage, message usage, device usage, visits (location), Ambient light	Demonstrated the feasibility of using Apple’s SensorKit framework to expand the range and quality of digital phenotyping data in mental health research, highlighting the improved granularity and potential clinical relevance of these new data streams. In this study, they focused on depression.
Cosgrove et al. [53]	Perspective	Tracking sensors	Critiques the ethical and human rights implications of digital phenotyping and sensor-embedded psychotropic drugs, warning that such technologies may reinforce coercion and compromise autonomy in mental health care. In this study, they focused on mood.
Moura et al. [54]	Review	GPS, accelerometer, app usage, sleep data, physical activity, mobility patterns	Reviewed 59 studies involving multimodal sensing for digital phenotyping of mental health. Highlighted the evolution, applications, and methodological challenges in using digital biomarkers for real-world clinical decision support. In this review study, they focused on mental health in general.
Cohen et al. [55]	Empirical study	Sleep duration patterns, digital activity	Demonstrated that smartphone-based digital phenotyping and mobile cognitive tasks show promising validity and cross-cultural applicability in assessing cognition and related behaviors among individuals with schizophrenia. In this study, they focused on schizophrenia.
Jilka et al. [56]	Perspective	GPS, accelerometer, app usage, and wearables as general sources of behavioral and physiological data	Advocates for broader adoption of digital phenotyping in mental health care; highlights its potential to improve ecological validity of assessments, enhance patient monitoring, and support real-world, data-driven interventions. In this study, they focused on mental health in general.
Lakhtakia et al. [57]	Empirical study	App usage, smartphone surveys, cognitive assessment games	Evaluated feasibility and acceptability of using smartphone digital phenotyping and cognitive tasks for monitoring symptoms in first-episode psychosis across India and the USA, demonstrating good engagement and preliminary clinical correlations. In this study, they focused on schizophrenia.
Song et al. [58]	Empirical study	Heart rate variability, sleep quality, physical activity	Demonstrated that wearable-derived sleep and activity data can predict daily depressive symptoms among vulnerable older adults, and showed preliminary benefits of individualized health feedback to users and caregivers. In this study, they focused on depression.
Currey et al. [59]	Empirical study	GPS, accelerometer, screen state, app usage	Demonstrated that digital phenotyping-based models predicting symptom improvement in students can generalize across two independent cohorts, supporting the external validity of sensor-derived behavioral features for mental health monitoring. In this study, they focused on stress, generalized anxiety disorder, loneliness, sleep quality, psychosis, depression, and anxiety.
Akbarialiabad et al. [60]	Perspective	GPS, accelerometer, social media, screen lock/unlock, call logs, camera, app usage, browser history, light sensor, sleep cycle, exercise, social interactions, heart rate	The commentary highlights the mental health risks and ethical challenges associated with the unregulated use of digital phenotyping and neuromarketing, particularly in vulnerable and low-resource populations. The authors call for greater regulation, transparency and the development of privacy-preserving technologies to safeguard mental health and personal autonomy. In this study, they focused on mental health in general.

### 3.2. Method of Designing ESFY

The ESFY, Expert System for Youth, system is designed to collect and interpret behavioral data derived from smartphones in order to detect early indicators of psychological distress. The underlying mechanism of the system is modeled after the insights from the scoping review and the framework proposed by Melcher et al. [1] (see Figure 1), which demonstrates how patterns of digital phenotypes can be systematically linked to the presence of significant psychological distress in users.

**Figure 3:**
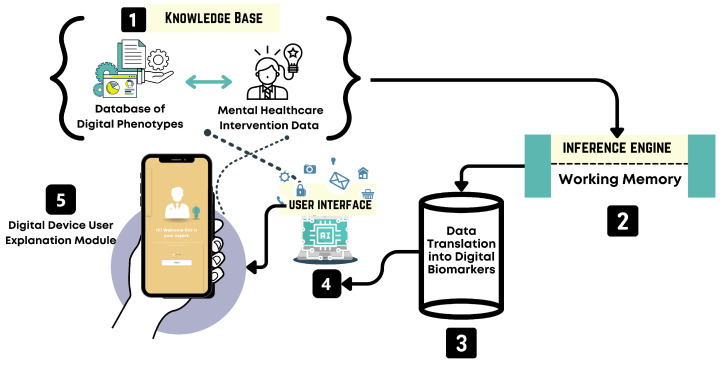
System architecture of the ESFY, illustrating the interaction between the user interface, knowledge base, and inference engine for real-time psychological distress detection. The architecture’s core components follow the design methodology outlined by [61]. The system comprises five key modules: (1) a knowledge base, which contains a curated database of digital phenotypes and mental healthcare intervention data; (2) an inference engine with a working memory component that processes inputs and draws conclusions based on stored knowledge; (3) a data translation module that converts raw device data into digital biomarkers; (4) a user interface that mediates communication between the user and the system, collecting behavioral data and delivering personalized outputs; and (5) a digital device user explanation module that presents feedback and insights directly to the user. Solid arrows indicate direct data flow, while dashed lines represent asynchronous or interpretive interactions, such as inference processes and user engagement dynamics.

The architecture of the system’s core components follows the knowledge-based system design methodology described by Gulavani and Kulkarni [61]. This methodology outlines a modular expert system architecture composed of a knowledge base, inference engine, explanation facility, and user interface, all supported by knowledge acquisition and engineering processes (see Figure 3).

The ESFY system provides each user with a personalized and secure digital workspace, facilitating the continuous collection and interpretation of smartphone-derived behavioral data. Through a user-friendly mobile interface, individuals can access real-time feedback about their mental health status, informed by both passive and active data streams such as physical activity, screen usage, and social interaction patterns. The system leverages a dynamic knowledge base, ensuring that recommendations and risk assessments are grounded in the latest evidence and tailored to the individual’s context. A central inference engine processes data as it is collected, drawing on both current and historical behavioral signals to identify patterns that may indicate early signs of psychological distress. Users are provided with timely, actionable insights and, where appropriate, guided interventions directly through the app interface. Privacy and user autonomy are emphasized throughout, with clear consent protocols and transparent data flows.

The ESFY system is designed as a modular, intelligent expert system for the early detection of psychological distress, leveraging digital phenotyping via passive smartphone data. The architecture is composed of three primary components: the user interface, the knowledge base, and the inference engine (see Figure 3). The user interface is designed for accessibility and engagement. Through this interface, users interact with the system in a manner that is intuitive and friendly, receiving both guidance and feedback. The user interface also serves as the entry point for collecting a diverse range of behavioral data streams from the user’s smartphone, such as activity patterns, social interactions, app usage, and other relevant digital phenotypes. The design prioritizes simplicity and privacy to maximize user adherence and trust. Central to the ESFY system is the knowledge base, which functions as a dynamic repository of domain-specific knowledge, behavioral rules, and evidence-based patterns associated with psychological distress. This module integrates findings from the scoping review and expert input. The knowledge base communicates directly with both the user interface and the inference engine, ensuring that all interactions and assessments are informed by the evidence. The inference engine forms the analytical core of the ESFY. It is responsible for processing incoming user data in real time, drawing upon the knowledge base to interpret behavioral signals and identify risk patterns. This component incorporates a working memory, which allows the system to maintain context and adapt recommendations dynamically as user behaviors and states evolve. By leveraging the inference engine, the ESFY is able to deliver highly personalized, context-aware feedback, supporting early intervention and ongoing monitoring. This modular and interconnected architecture ensures that the ESFY remains adaptable, scalable, and responsive to individual needs while maintaining the privacy required for digital mental health applications. Together, these components create a cohesive system capable of translating complex digital traces into actionable mental health insights.

The ESFY system incorporates a suite of carefully designed features aimed at supporting the early detection and self-management of psychological distress. These features are informed by the insights gained from our scoping review of recent digital phenotyping research, as well as the foundational framework proposed by Melcher et al. [1]. Drawing from evidence-based practices and the latest findings in the field, each component of the ESFY is tailored to facilitate user engagement, enhance self-awareness, and enable timely interventions. In this section, we describe the primary functionalities and technical components of the ESFY, highlighting how each is intended to promote mental health monitoring and empower users to take proactive steps toward well-being.

#### 3.2.1. Daily Prognosis and Insights Hub

**Figure 4:**
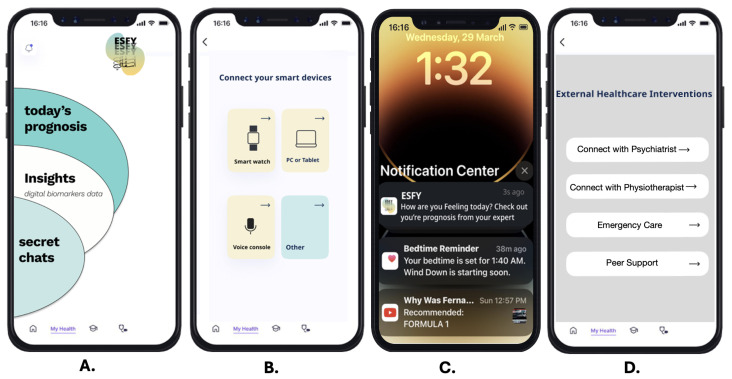
User interfaces of key features of the ESFY system: (**A**) home screen with daily prognosis, behavioral insights, and chat access; (**B**) multi-device integration for data collection; (**C**) proactive user notifications; (**D**) direct access to external healthcare interventions.

The home screen presents a holistic snapshot of the user’s current well-being, summarizing insights and risk levels based on real-time digital phenotyping data (Figure 4A). By featuring “today’s prognosis,” users receive an immediate, actionable overview of their mental health status for that day. The “Insights” section surfaces interpretable analytics from collected digital biomarkers, such as activity patterns or sleep quality, empowering users to better understand the connection between their digital behaviors and psychological health.

#### 3.2.2. Multi-Device Integration Panel

The ESFY supports seamless data collection through multi-device integration, as showcased in the device connection interface (Figure 4B). Users can pair the system with a range of smart devices, including smartwatches, PCs, or tablets. This feature enables richer, multi-modal data collection by leveraging wearables and IoT devices to gather physiological signals (e.g., heart rate from a smartwatch) alongside digital activity from other platforms. Such extensibility increases the system’s accuracy in monitoring behavioral and physiological phenotypes while enhancing user flexibility and inclusivity.

#### 3.2.3. Proactive Notifications and Feedback

The ESFY notification system is designed to engage users proactively, delivering real-time updates and reminders directly to the device’s notification center (Figure 4C). Sample notifications include prompts to check their daily prognosis, bedtime reminders, or recommended health actions. This feature ensures that critical mental health insights and behavior recommendations are delivered promptly, promoting adherence to self-care routines and maximizing opportunities for early intervention.

#### 3.2.4. External Healthcare Interventions

Recognizing the importance of integrated care, the ESFY provides users with direct pathways to external healthcare support (Figure 4D). The interface features clear options to connect with a psychiatrist, physiotherapist, emergency care, or peer support. By facilitating seamless referrals and crisis intervention, this feature ensures that users who exhibit signs of psychological distress are able to access appropriate professional or community resources promptly.

#### 3.2.5. Health Stats Overview

**Figure 5:**
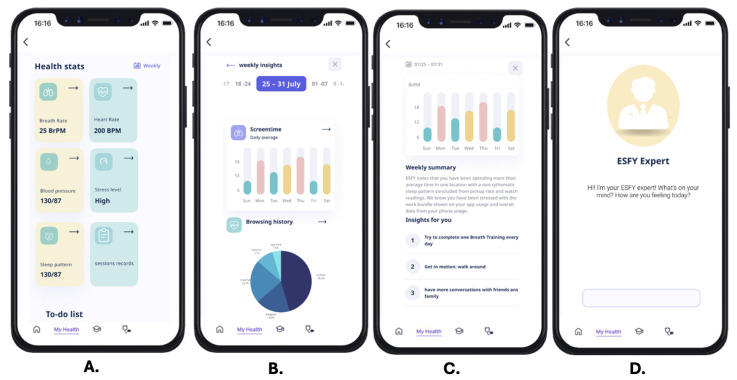
Key ESFY system interfaces: (**A**) Health Stats dashboard summarizing physiological and behavioral data; (**B**) Weekly Insights with visual analytics of screen time and browsing history; (**C**) Automated Weekly Summary and actionable health recommendations; (**D**) ESFY Expert Conversational Interface for personalized user support.

The Health Stats screen serves as a personalized health dashboard, providing users with a concise summary of their key physiological and behavioral metrics (Figure 5A). Displayed indicators include breath rate, heart rate, blood pressure, stress level, and sleep pattern. Each metric is visually distinguished by color-coded cards and accompanied by clear icons and current values, making complex data accessible at a glance. The inclusion of a weekly toggle allows users to view trends over time, while the integrated to-do list feature supports the setting and tracking of personal health goals. This interface is central to promoting user self-awareness and proactive health management through digital phenotyping.

#### 3.2.6. Weekly Insights Analytics

The weekly insights analytics module provides users with interactive visualizations of their digital behaviors (Figure 5B). Different data visualizations allow users to track daily average screen time across the week, enabling users to identify trends, spikes, or irregularities in their device usage. Complementing this is a breakdown of browsing history, detailing the proportion of time spent on major platforms such as YouTube, Instagram, Snapchat, and more. By surfacing these insights, the ESFY empowers users to reflect on their digital habits and their potential impact on psychological well-being, laying the groundwork for positive behavior change.

#### 3.2.7. Automated Weekly Summary and Actionable Insights

This screen features an automated weekly summary generated by the ESFY system, synthesizing user data into a plain-language overview and tailored recommendations (Figure 5C). The summary describes observed behavior patterns, such as time spent in one location, sleep rhythm irregularities, or stress-related digital activity. The system provides specific, actionable insights, such as completing daily breath training, increasing physical activity, or having more conversations with friends and family. By translating raw digital phenotyping data into simple, personalized suggestions, this feature bridges the gap between data collection and real-world mental health improvement.

#### 3.2.8. ESFY Expert Conversational Interface

The ESFY Expert Conversational Interface features a conversational AI assistant designed to offer personalized guidance, emotional support, and daily mental health check-ins through a minimal and user-friendly interface (Figure 5D). By initiating open-ended reflections and greeting users in a nonjudgmental tone, the virtual assistant helps foster a private, stigma-free space that encourages honest self-expression and sustained engagement. Drawing from insights in our scoping review, which indicate that many youth prefer anonymous interactions over direct engagement with mental health professionals [62], the system includes a dedicated “Secret Chat’’ functionality. This feature is powered by an AI-driven chatbot, not connected to any other users, and is available 24/7 to simulate empathetic, reflective conversations. It offers users a safe outlet for emotional disclosure, mood processing, and stress de-escalation during moments when professional support may be unavailable or inaccessible. This design choice aligns with prior work on the therapeutic potential of anonymous digital interactions [63] and serves as a just-in-time, low-barrier entry point for early distress intervention.

### 3.3. User Study Method

To evaluate the ESFY system and gain a deeper understanding of user experience with its mental health support features, we conducted a mixed-methods user study. The primary objectives of this study were to assess the usability of the ESFY system and to gather insights into its potential effectiveness for the early detection and self-management of psychological distress. The study was conducted in accordance with the Declaration of Helsinki and approved by the Institutional Review Board of the University of Jeddah (UJ-REC-344) on 8 May 2025.

Written informed consent was obtained from all subjects involved in the study.

#### 3.3.1. Study Procedure and Design

The user study was conducted remotely using the Maze usability testing platform and was organized into three distinct phases to capture a comprehensive picture of user experience with the ESFY prototype.

1. Completion of Prototype Tasks: Participants were asked to complete a predefined set of tasks that simulated typical interactions with the ESFY system. These tasks were designed to reflect real-world use cases, such as onboarding, reviewing personalized mental health feedback, and interacting with behavioral insights generated by the system. As participants navigated through each task, they moved sequentially from one screen to another, making choices and utilizing the app’s core features to achieve specific goals. Throughout the study, multiple usability and performance metrics were systematically recorded, including the average duration spent on each task, average misclick rate, and task drop-off rates. We also tracked the number of participants who completed each mission by following the expected path (direct success) versus those who achieved completion through alternative navigation paths. Additionally, we computed the average success rate across all tasks and measured the average time each participant required to complete a given mission.
2. Follow-Up Questionnaires: To complement these quantitative metrics, participants were prompted with open-ended questions at the conclusion of their interactions, allowing them to provide qualitative feedback on their experiences, challenges, and suggestions for improvement.
3. NASA TLX Survey: To assess cognitive workload, participants completed the NASA TLX [64], which measures mental demand, effort, frustration, and other usability factors when interacting with the ESFY.

#### 3.3.2. Participants

A total of (n=36) participants took part in the user study. Participants were recruited from a university population in Saudi Arabia using the widely adopted social media platform WhatsApp. The sample consisted of 2 males and 34 females, with ages ranging from 18 to 24 years (M=21.0, SD=1.73). The predominance of female participants can be attributed to both the gender of the principal investigator, who is female, and the structural organization of the university, which maintains separate branches for male and female students. As a result, recruitment efforts and access to participants were naturally concentrated within the female branch, leading to a higher representation of female students in the sample. We highlight this in the limitations section.

## 4. Results

This section presents the findings from the different phases of the study: the scoping review and the user study. The scoping review synthesized literature at the intersection of psychological distress and digital phenotyping, with a particular focus on youth populations. The findings are presented in two parts: the first outlines how psychological distress is conceptualized, identified, and addressed in existing research; the second examines the use of digital phenotyping techniques for mental health monitoring, highlighting their applications, challenges, and potential for early detection. These findings informed the design and development of the ESFY prototype. The second part of this section details the results of a mixed-method user study conducted to evaluate the ESFY system, focusing on its functionality, user experience, and real-world applicability. Together, these results provide insight into both the current state of research and the practical potential of digital phenotyping tools for early detection of psychological distress among youth.

### 4.1. Scoping Review Results: Psychological Distress

This subsection reviews how psychological distress is defined, measured, and studied in the existing literature. It summarizes the key psychological constructs involved (e.g., anxiety, depression, loneliness), common screening tools and self-report measures, and demographic or contextual factors influencing distress. The review also identifies gaps in early detection strategies and limitations in traditional assessment methods that digital approaches aim to address.

#### 4.1.1. Stress

A number of studies directly examined the use of digital phenotyping for stress detection. Choi et al. [40] conducted a systematic review of smartphone sensors and found strong evidence that data such as GPS, microphone, light sensor, accelerometer, phone locks/unlocks, and other modalities can effectively identify behavioral patterns associated with stress in nonclinical populations. Expanding on this, Egger et al. [45] proposed and evaluated a real-time stress assessment framework that integrated multiple passive data streams, including GPS, accelerometer, screen state, call logs, and app usage, demonstrating that multimodal sensing can significantly enhance early stress detection and intervention. Similarly, Shvetcov et al. [46] developed and validated a machine learning pipeline utilizing GPS, accelerometer, and app usage data, successfully predicting stress levels among university students through passive smartphone sensing. Collectively, these studies highlight that digital phenotyping provides a reliable, scalable method for capturing stress-related behaviors and reinforces the value of combining multiple passive sensing modalities to enable earlier and more accurate identification of psychological distress.

#### 4.1.2. Anxiety

Several studies explicitly addressed anxiety using digital phenotyping modalities. Jacobson et al. [41] demonstrated that accelerometer, call logs, and SMS data could predict social anxiety severity using machine learning models. Similarly, Cohen et al. [3] showed that combining geolocation, accelerometer, screen state, and active survey responses enabled anomaly detection of mood and anxiety symptom changes in clinical populations. Zhang et al. [4] further expanded this approach by analyzing wearable-derived physical activity, heart rate, and sleep patterns, successfully identifying behavioral markers associated with both depression and anxiety in a large-scale sample. Nguyen et al. [43] applied machine learning to pseudo-passive, survey-derived data, achieving classification of anxiety severity during the COVID-19 pandemic. Additionally, Kang et al. [44] introduced a hybrid model that combined app usage, smartphone activity logs, and wearable sensor data to assess mood and anxiety at a large scale. Together, these studies demonstrate that anxiety symptoms can be reliably detected through a variety of passive and active digital data streams, particularly when multimodal inputs are integrated to capture the complexity of behavioral and emotional states.

#### 4.1.3. Loneliness

Melcher et al. [1] provide important insights into the use of digital phenotyping for assessing loneliness among college students. Their clinical review highlights that social information streams, particularly call logs and SMS activity, were captured in approximately half of the studies reviewed, enabling objective measurement of social engagement and potential isolation. Some studies also combined communication data with location tracking to infer patterns of social behavior, offering richer insights into loneliness and social isolation. Other passive sensing modalities, such as Bluetooth and microphone data, were discussed as promising for detecting nearby devices or ambient conversations, which could indicate in-person social interactions. Additionally, screen time patterns were examined, with reduced or irregular usage potentially serving as markers of social withdrawal. While Melcher et al. [1] emphasize the feasibility and acceptability of collecting these digital signals among college populations, they also highlight the need for standardized protocols and larger sample sizes to fully realize the potential of digital phenotyping for unobtrusive loneliness detection and personalized mental health interventions.

#### 4.1.4. Depression

Several studies directly examined the application of digital phenotyping for detecting depression. Choi et al. [40] found that a comprehensive range of smartphone sensors, including GPS, microphone, light sensor, accelerometer, phone locks/unlocks, call logs, Bluetooth, Wi-Fi, keyboard activity, SMS/email, app usage, screen activity, and gyroscope data, can effectively identify behavioral patterns associated with mild depressive symptoms. Expanding this work, Zhang et al. [4] analyzed wearable-derived physical activity, heart rate, and sleep patterns in a large sample of participants, successfully identifying markers of depression (and anxiety) and demonstrating the scalability of wearable-based digital phenotyping. Similarly, Song et al. [58] conducted a pilot study with older adults, showing that daily depressive symptoms could be predicted through wearable-derived heart rate variability, sleep quality, and physical activity data, offering meaningful, individualized feedback. Together, these studies reinforce the potential of both smartphone and wearable technologies to provide continuous, scalable, and personalized monitoring of depressive symptoms across diverse populations.

#### 4.1.5. Other Psychological Distress Types

In addition to stress, anxiety, loneliness, and depression, several studies in this review addressed additional psychological distress types and related constructs using digital phenotyping methods. A number of papers examined broad mental health constructs and general symptom monitoring focusing on the feasibility, challenges, or broad application of digital phenotyping for mental health assessment, remote monitoring, symptom changes, or the identification of digital biomarkers for real-world clinical support [4,37,45,53]. Mood was another construct explicitly examined. Cohen et al. [3] and Kang et al. [44] demonstrated that passive and active smartphone data, including geolocation, accelerometer, screen state, survey responses, and app usage, can be used to monitor significant mood symptom changes across clinical and community cohorts. Cognitive impairment and related symptoms were investigated also. Cohen et al. [55] showed that smartphone-based digital phenotyping and mobile cognitive tasks have promising validity for assessing cognition and related behaviors among individuals with schizophrenia. Lakhtakia et al. [57] evaluated smartphone digital phenotyping and cognitive tasks for monitoring symptoms in first-episode psychosis, demonstrating good engagement and clinical correlations. Psychosis and schizophrenia were directly addressed in both Cohen et al. [55] and Lakhtakia et al. [57], where the integration of smartphone data streams with cognitive assessment tasks was found to be feasible and potentially valuable for symptom monitoring in these severe mental illnesses. Finally, symptom improvement as an outcome was validated in other studies. Hays and Torous [49] and Currey et al. [59] both demonstrated that digital phenotyping-based models could predict symptom improvement and guide personalized interventions, supporting the external validity and clinical utility of these approaches for ongoing mental health monitoring.

#### 4.1.6. Summary

The collective evidence from the reviewed literature demonstrates that digital phenotyping, through the analysis of both smartphone and wearable sensor data, has emerged as a powerful approach for the early detection and monitoring of psychological distress, particularly stress, anxiety, and depression. A consistent finding across studies is the versatility and sensitivity of passive data streams, such as GPS for mobility patterns, accelerometer for physical activity, app usage for digital behavior, and communication logs for social engagement, in capturing subtle changes associated with mental health fluctuations. Beyond the primary focus on stress, anxiety, and depression, this review also revealed the expanding scope of digital phenotyping research into broader mental health and neuropsychiatric domains. A subset of studies examined the application of digital phenotyping to mood instability, cognitive impairment, psychosis, and overall symptom improvement, demonstrating the adaptability of these methodologies to diverse clinical populations. Notably, several empirical studies validated the clinical utility of digital phenotyping for ongoing monitoring, risk prediction, and the delivery of personalized interventions, signaling increasing maturity and translational potential in the field. Overall, the literature consistently highlights the value of integrating diverse data streams, combining behavioral, physiological, and subjective data, to achieve more accurate, scalable, and timely identification of psychological distress.

### 4.2. Scoping Review Results: Digital Phenotyping

This subsection focuses on how digital phenotyping has been applied in mental health research. It highlights the types of behavioral and device-derived data most commonly used (e.g., screen time, sleep patterns, mobility, communication logs), the analytical techniques employed (e.g., machine learning, passive sensing), and the ethical and technical challenges reported in the literature. The review also identifies patterns in how digital phenotyping contributes to the early identification of psychological distress and emphasizes its potential scalability and accessibility for youth mental health interventions.

#### 4.2.1. GPS/Geolocation

GPS and geolocation data were commonly used across studies to monitor individuals’ mobility patterns, daily routines, and location variability, key behavioral indicators for psychological distress. Changes in mobility, such as reduced travel range or disruptions to habitual movement patterns, were often linked to elevated symptoms of stress, anxiety, and depression. For instance, Choi et al. [40] systematically reviewed how reduced movement and irregular geospatial behavior correlated with higher levels of psychological distress. Similarly, Mendes et al. [23] incorporated GPS-derived features into a supervised machine learning framework to predict stress in university students, demonstrating the practical utility of location-based sensing. In clinical populations, GPS data also supported the identification of mood and anxiety symptom changes, with sudden shifts in movement patterns serving as early indicators of distress [3]. Collectively, these findings highlight GPS data as a foundational digital phenotype, enabling scalable and unobtrusive monitoring of mental health fluctuations in both general and clinical settings.

#### 4.2.2. Accelerometer

The accelerometer was frequently employed to quantify physical activity levels and movement patterns, which serve as valuable indicators of psychological distress. Studies have shown that reduced physical activity and disruptions to daily movement routines may reflect underlying symptoms of anxiety, depression, or stress. For example, Jacobson et al. [41] demonstrated that lower activity levels, as captured by smartphone accelerometer data, could effectively predict social anxiety severity in young adults. Similarly, large-scale analysis using wearable data revealed that decreased daily activity was significantly associated with both depressive and anxiety symptoms [4]. These findings collectively support the role of accelerometer data as a reliable and non-invasive digital phenotype for capturing behavioral markers relevant to a broad spectrum of psychological distress.

#### 4.2.3. App Usage

App usage data provided valuable insight into participants’ digital routines, screen engagement, and behavioral changes. Several studies demonstrated that fluctuations in app usage patterns could serve as proxies for mental health states. For instance, Currey et al. [59] showed that app usage features could predict symptom improvement and guide personalized interventions among college students experiencing depression and anxiety. Similarly, Nguyen et al. [43] used app usage logs alongside smartphone activity data to classify anxiety severity and assess mood changes, particularly during high-stress periods such as the COVID-19 pandemic. Additionally, app usage was incorporated in real-time stress detection models alongside other passive data streams like GPS and screen state [45]. These findings collectively emphasize the potential of app usage data as a dynamic and scalable indicator for detecting shifts in psychological distress and informing individualized mental health support.

#### 4.2.4. Screen Activity

Screen activity, including phone unlock frequency, duration of screen time, and irregular device engagement, was used across multiple studies to infer behavioral patterns linked to psychological distress. Cohen et al. [3] found that inconsistent or frequent phone use was associated with heightened anxiety and mood symptom fluctuations. Similarly, Melcher et al. [1] noted that reduced or irregular screen activity among college students could signal social withdrawal and loneliness. In older populations, Currey and Torous [48] showed that disruptions in daily screen engagement, when combined with wearable-derived data, were predictive of depressive symptoms. Furthermore, Egger et al. [45] integrated screen state into a real-time stress monitoring framework, highlighting its potential to signal shifts in daily routines. Collectively, these studies suggest that screen activity serves as a passive, yet sensitive, indicator of emotional and cognitive well-being, particularly when analyzed alongside other digital phenotypes.

#### 4.2.5. Call Logs

Call log data served as a proxy for social interaction, with frequency, duration, and timing of calls used to infer levels of social engagement or withdrawal. Jacobson et al. [41] found that fewer and shorter calls were associated with higher levels of social anxiety, suggesting that reduced phone communication may reflect avoidant behavior. Melcher et al. [1] similarly emphasized that diminished call activity among college students correlated with loneliness and social isolation. Choi et al. [40] further highlighted the relevance of call logs in detecting stress and depression by identifying deviations in regular communication behavior. Additionally, Egger et al. [45] incorporated call logs in a real-time monitoring framework for stress, demonstrating how such passive data can support continuous mental health assessment. These studies collectively underscore the value of communication metadata as a non-intrusive indicator of psychological well-being, particularly in detecting socially rooted forms of distress.

#### 4.2.6. SMS and Email

SMS and email logs provided an additional layer of insight into users’ communication behaviors and potential social well-being. Jacobson et al. [41] identified that fewer sent text messages were significantly associated with higher levels of social anxiety, suggesting that decreased digital communication may serve as an indicator of avoidance or interpersonal withdrawal. Similarly, Melcher et al. [1] emphasized that patterns of SMS and email activity, such as reduced frequency or irregular communication, could be utilized as passive markers of loneliness and social isolation, mirroring the patterns observed through call log analysis. Although SMS and email were not the primary focus in most studies, these modalities contribute meaningfully to the broader landscape of digital phenotyping by offering unobtrusive, low-burden methods for inferring changes in social connectedness, a critical dimension of mental health.

#### 4.2.7. Bluetooth, Wi-Fi, and Proximity Sensing

Although less frequently emphasized than other digital phenotypes, Bluetooth and Wi-Fi signals were explored in a few studies for their potential to infer real-world social proximity and physical co-location with others. For instance, Choi et al. [40] discussed Bluetooth and Wi-Fi as passive sensing modalities capable of detecting nearby devices or shared network environments, which can serve as proxies for in-person social interaction. Similarly, Melcher et al. [1] highlighted the promise of these modalities in identifying degrees of social connectedness or isolation. While these proximity indicators are theoretically valuable, especially in contexts where physical presence is a marker of social well-being, most reviewed studies treated them as prospective features rather than established predictors. As such, they were more often framed as future avenues for research than fully validated tools.

#### 4.2.8. Light Sensor and Microphone

Light sensors and microphone data were considered in a few studies as valuable supplementary data streams for contextualizing daily routines and social environments. Melcher et al. [1] discussed the potential of microphone data to detect ambient conversation, which could offer a passive measure of real-world social engagement and help infer loneliness or social withdrawal. Similarly, Mendes et al. [23] referenced both light sensors and ambient data collection as relevant to understanding circadian rhythm disruptions, activity timing, and broader behavioral contexts. Akbarialiabad et al. [60] further emphasized the growing use of ambient interaction tracking, implicitly including light and audio cues, in digital mental health tools, while also cautioning against their use without appropriate ethical oversight. Although not yet widely validated as core predictors, these sensing modalities show promise for enhancing the granularity of behavioral inference in future digital phenotyping systems, particularly when triangulated with other passive data.

#### 4.2.9. Physical Activity, Heart Rate, and Sleep Patterns

Several studies utilized wearable-derived physiological signals, particularly physical activity, heart rate, and sleep metrics, as indicators of psychological distress. Zhang et al. [4] demonstrated that lower levels of physical activity, disrupted sleep duration, and changes in heart rate variability were significant predictors of both depression and anxiety across a large-scale, longitudinal cohort. Similarly, Currey and Torous [48] validated the use of wearable sensors to detect daily fluctuations in depressive symptoms among older adults, highlighting the value of heart rate variability and sleep quality as sensitive, day-to-day markers of mental well-being. Building on this, Song et al. [58] implemented a community-based monitoring platform that used sleep and activity data to assess depressive symptoms in geriatric populations, confirming the feasibility of passive sensing in real-world, older adult settings. These physiological phenotypes offer a reliable, passive means of capturing psychological state shifts over time, especially when integrated with behavioral data such as app usage or screen activity.

#### 4.2.10. Self-Reported Data

Active digital surveys were integrated with passive sensing in several studies to contextualize and validate behavioral inferences. Cohen et al. [3] utilized self-reported measures of mood, anxiety, or loneliness to ground passive sensor data such as location, activity, and communication patterns in participants’ subjective experiences. These inputs were typically gathered through ecological momentary assessments or daily check-ins delivered via smartphone apps. Similarly, Nguyen et al. [43] applied machine learning models to pseudo-passive, survey-derived data, collected during the COVID-19 pandemic, to classify anxiety severity, highlighting the value of structured self-report as an analytic feature. In ref. [38], researchers combined passive smartphone sensing with active survey responses to monitor symptom fluctuations and better understand the relationship between digital behaviors and mental health. Across these studies, self-reported data provided essential validation anchors and interpretive depth, strengthening the reliability and relevance of digital phenotyping outputs.

#### 4.2.11. Summary

This review highlights the diverse and complementary roles of digital phenotyping modalities in detecting psychological distress. GPS, accelerometer, app usage, and screen activity emerged as the most empirically supported signals, consistently linked to stress, anxiety, depression, and loneliness. Additional data streams, including call logs, SMS/email activity, Bluetooth-based proximity sensing, and wearable-derived physiological metrics such as heart rate and sleep quality, further expanded the behavioral landscape captured across studies. Light sensor and microphone data were also noted as promising for contextualizing activity patterns and social environments. The integration of active self-reports with passive data proved crucial for enhancing the validity and interpretability of findings. Collectively, these insights confirm that combining multiple behavioral and physiological modalities can significantly strengthen early detection capabilities. Building on the findings of this scoping review and guided by the conceptual framework proposed by Melcher et al. [1] (Figure 1), we designed and developed the ESFYprototype system to operationalize these insights into a functional, youth-focused mental health support platform.

### 4.3. User Study Results

**Figure 6:**
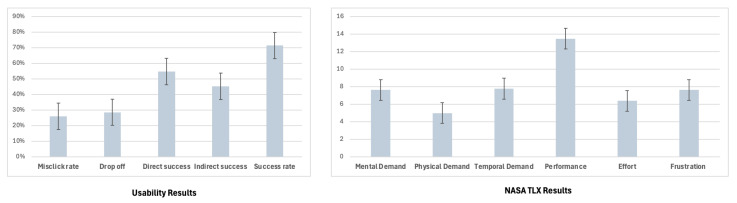
The left chart displays key usability metrics, including misclick rate, drop-off rate, direct and indirect task success rates, and overall success rate. The right chart presents the NASA TLX results across six workload dimensions, mental demand, physical demand, temporal demand, performance, effort, and frustration. Error bars represent standard error of the mean for each metric.

Analysis of task performance within the ESFY system revealed that participants were generally able to complete assigned activities efficiently, with an average duration of 29.6 s per task. These results indicate that most users could navigate the core features of the app quickly. However, efficiency must be balanced with accuracy and ease of use to ensure a positive experience. Despite the relatively fast task completion times, challenges in interface clarity were evident. The average misclick rate was 26.0%, suggesting that over one in four interactions did not proceed as intended. This rate of input errors may reflect ambiguous interface elements or a learning curve associated with the ESFY system. Furthermore, the participant drop-off rate stood at 28.6%, highlighting a substantial proportion of users who disengaged before completing all tasks (Figure 6). Together, these findings point to areas where additional refinement of the user journey and guidance is warranted to reduce confusion and attrition.

Examining the pathways to success, 54.76% of users completed the missions using the expected, direct path, while a notable 45.24% relied on alternative navigation strategies. This near-even split indicates that while the main workflow is functional for a majority, a significant number of users either intentionally or inadvertently took detours, possibly as a result of unclear prompts or multiple available routes. The average overall success rate across all tasks was 71.4%, demonstrating that most participants achieved their goals, but that a meaningful portion encountered obstacles. These metrics collectively highlight the importance of optimizing both the clarity and flexibility of the ESFY system to maximize usability, engagement, and successful task completion for all users.

#### 4.3.1. Cognitive Workload

Participants generally reported moderate cognitive demand while interacting with the system (Figure 6). Among the six dimensions measured, performance received the highest mean score, indicating that participants perceived a strong sense of accomplishment and effectiveness in task completion. Mental demand and temporal demand were rated at intermediate levels, suggesting that while participants were cognitively engaged and mindful of time constraints, these factors were not overwhelming. Physical demand and effort both received relatively lower scores, reflecting the app’s low physical interaction requirements and the reasonable amount of effort needed to complete tasks. Frustration was also rated at a moderate level, implying that although users encountered some challenges or confusion, these did not lead to excessive dissatisfaction or disengagement. Taken together, these findings indicate that the ESFY prototype presents a manageable cognitive workload for users, with most demand dimensions remaining well below maximum levels. The relatively high performance ratings, alongside moderate mental and temporal demands, suggest that the interface enables sustained engagement for the target users.

#### 4.3.2. Usability and Aesthetics

**Figure 7:**
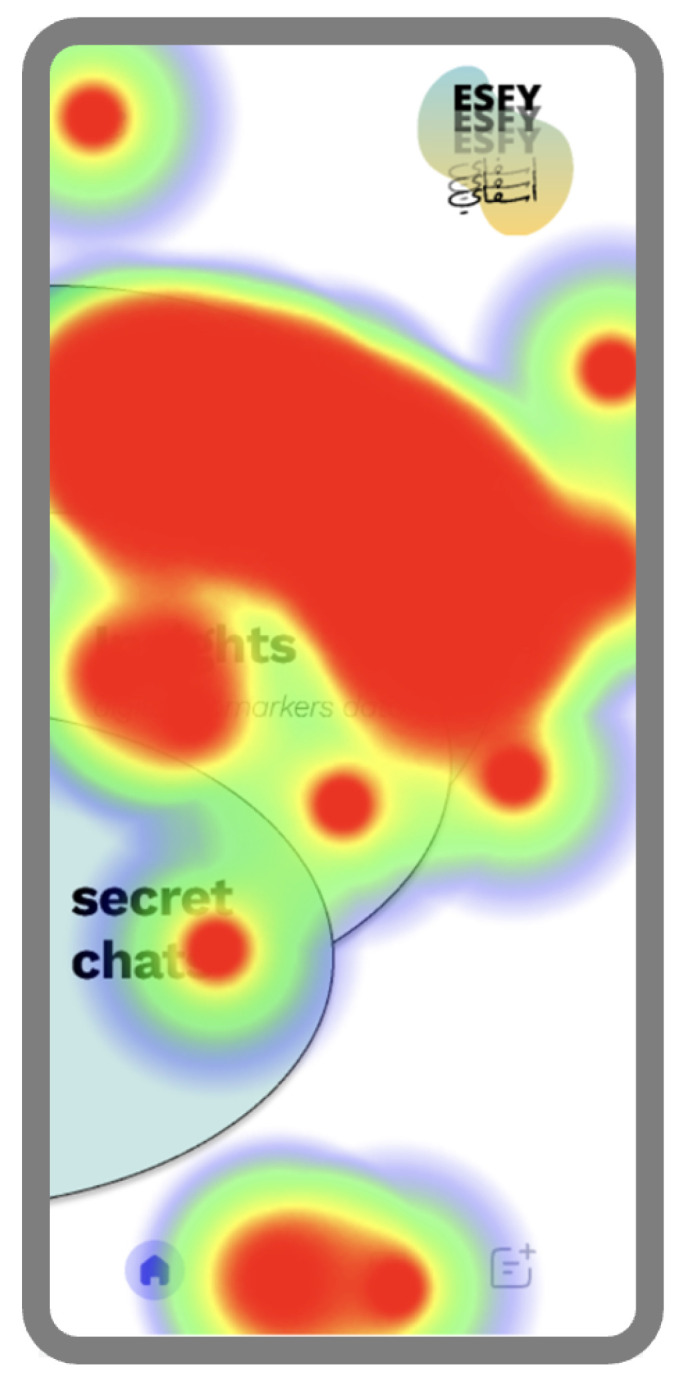
User interaction heatmap showing areas of highest attention on the ESFY main screen.

User perceptions of the ESFY system were notably positive with regard to usability and aesthetics. Several participants highlighted the app’s intuitive design and visual appeal, with one stating, *“It is a very pretty and easy to use app.” [P26]*. Another echoed this sentiment, noting, *“The current offerings meet my needs effectively. I find the app user-friendly and efficient.” [P6]*. Such feedback indicates that the system’s emphasis on clear navigation and attractive interface elements contributed to an accessible and engaging user experience, an important factor for encouraging adoption. Ease of self-monitoring and the psychological benefits of digital health tracking also emerged as recurring themes. One participant remarked, *“Makes me keep track of my health in an easy way, and makes me feel better to have everything I feel or do saved.” [P29]*. This response suggests that the ESFY not only simplifies the process of tracking daily well-being but may also provide users with a sense of control and reassurance through accessible, centralized records of their health behaviors and feelings. At the same time, openness to digital health tools was nuanced and context-dependent. As one participant observed, *“I would not have any problem with those applications if it’s needed.” [P19]*. This insight reflects an underlying willingness to adopt such systems when they are perceived as relevant or necessary while also implying that personal motivation and individual circumstances are significant determinants of engagement. Heatmap analysis of the ESFY system (Figure 7) revealed distinct patterns in user attention and interaction. In general, participants were particularly drawn to features that were clearly marked and easy to understand when navigating the app.

#### 4.3.3. Clarity and Perceived Value

While many users found the ESFY system accessible and beneficial, several participants voiced concerns regarding clarity and overall value. One participant commented, *“Wasn’t sure how to use the simulation, a bit confusing at times,” [P22]* indicating that certain aspects of the system’s workflow or instructions may have lacked sufficient guidance, leading to moments of uncertainty or hesitation during task completion. In addition, a minority of participants questioned the utility of the app, with one stating, *“I think they are not very helpful,” [P14]*, and another expressing, *“I don’t think they are benefiting honestly.” [P16]*. These responses highlight that while the app succeeded in delivering a positive experience for many, there remains a subset of users for whom the current implementation did not meet expectations for perceived usefulness or ease of use. Addressing these concerns will require refining onboarding processes, enhancing in-app instructions, and further clarifying the practical value of digital phenotyping insights in supporting mental health.

#### 4.3.4. Integration with Healthcare

Some participants offered reflections on the appropriate target audience and role of digital health applications like the ESFY, emphasizing both potential benefits and important boundaries. One user commented, *“It’s a wonderful idea and the target audience should be people who need their health monitored closely, such as chronically ill patients and elderly people (although the older generations would probably be much more hesitant towards the idea of such apps).” [P8]*. This observation aligns with our previous research indicating ongoing challenges with the adoption of mHealth applications among older adults in Saudi Arabia, often due to technological hesitancy or unfamiliarity [6]. Additionally, participants highlight the need for digital tools to act as a supplement, rather than a substitute, for clinical expertise, with one stating, *“While these tools can be helpful, they should complement professional medical advice, not replace it.” [P23]*. Together, these responses reinforce the importance of both tailoring digital solutions to user readiness and maintaining a clear distinction between app-based guidance and formal healthcare interventions.

#### 4.3.5. Accuracy and Reliability

Concerns regarding the accuracy of digital health applications were evident in participant feedback. One user described the system as *“helpful, questionable in terms of accuracy,” [P30]*, while another stated, *“They are good because I can track and monitor my health however I feel like it is not accurate.” [P1]*. These comments highlight that, despite the perceived utility and convenience of the ESFY system for health monitoring, skepticism remains about the reliability of the information provided. This perception may influence users’ willingness to fully trust or act upon the app’s recommendations, suggesting a need for greater transparency about how insights are generated and, where possible, ongoing validation of the underlying digital phenotyping algorithms.

#### 4.3.6. Privacy and Transparency

Privacy concerns were a central theme in participant feedback, reflecting a broader societal sensitivity to the handling of personal health data. Several users explicitly stated that their willingness to adopt and use digital health applications like the ESFY is conditional on the robust protection of their privacy. As one participant remarked, *“They are great, as long as privacy is maintained,” [P35]*, while another emphasized, *“It’s important to be cautious of privacy concerns, as health data is sensitive.” [P23]*. These perspectives highlight the need for clear communication about how data is stored, who has access to it, and the specific measures in place to safeguard user confidentiality. The sensitivity surrounding health information means that any ambiguity or lack of transparency in data management can become a significant barrier to user trust and engagement.

Beyond privacy alone, users also expressed a strong preference for transparency and granular control over their data. One participant noted, *“I think such apps can be extremely useful and helpful as long as they are transparent about the data they collect and allow for customization of such features either through selected questions for each type of data or by only using data imputed by the user.” [P5]*. Another participant articulated skepticism about app adoption in the absence of institutional oversight, stating, *“I’m honestly quite skeptical about using such apps if they are not connected to some sort of healthcare facility because I’d be concerned about my privacy or where the data being collected would be used.” [P4]*. Together, these responses highlight the importance of giving users meaningful choices about what data is collected and how it is used. Transparency and user empowerment, especially regarding data collection and sharing, are critical for fostering a sense of control and trust, both of which are prerequisites for widespread acceptance and effective use of digital phenotyping technologies in mental health.

#### 4.3.7. Suggestions for Future Features

Participants also offered valuable suggestions for future enhancements to the ESFY system, reflecting both emerging expectations and opportunities to expand the app’s utility. One user highlighted the appeal of a *“voice interaction as an agent to have a conversation with (the expert),” [P30]* suggesting that the integration of voice-based AI could create a more natural, engaging, and accessible support experience. Another participant recommended the addition of an *“App lock feature so the user can take a mental break from social media,” [P12]* highlighting the growing recognition of the need for digital wellness tools that empower users to manage and moderate their screen time. Finally, the idea of a daily diary feature was raised. *“Diary, where each day there’s a specific notes app for, so people could record how they felt or what they experienced in a certain day, and then the app could check if there’s any correlation between certain note entries and psychological or physical effects.” [P16]*. This suggestion points to the potential value of personalized, longitudinal self-reflection within the app, an approach that could enhance both self-awareness and the ability to detect patterns relevant to mental health outcomes. Collectively, these recommendations highlight promising directions for future development that align closely with the needs and expectations of users.

## 5. Discussion

This research adopted a comprehensive, three-phase approach to advance the early detection of psychological distress using digital phenotyping. Through a scoping review, the project first mapped the current landscape of digital phenotyping modalities, relevant behavioral markers, and their associations with key mental health outcomes. Insights from this synthesis directly informed the subsequent design and development of the ESFY system, a user-centered platform leveraging both passive and active data to support youth mental health. Finally, an empirical evaluation of the ESFY prototype, incorporating both quantitative usability metrics and qualitative user feedback, provided critical validation and surfaced practical challenges and opportunities for system improvement. By systematically linking evidence from the literature to technical design decisions and real-world user experiences, this study offers an integrated perspective on how digital phenotyping tools can be effectively developed and deployed for scalable psychological distress detection. This aligns with previous work advocating for scalable, real-time monitoring of mental health using mobile data streams [16,17].

The scoping review revealed a rapidly evolving field where digital phenotyping, particularly through smartphones and wearables, shows considerable promise for early detection and monitoring of psychological distress. Most contemporary research emphasizes the importance of integrating multiple data streams, such as location, activity, app usage, and communication patterns, to capture nuanced indicators of stress, anxiety, depression, and, to a lesser extent, loneliness. This is consistent with findings from Kostopoulos et al. [10] and Winslow et al. [11], who demonstrated that multi-modal behavioral signals significantly improve stress and depression detection. A notable insight is the growing value placed on combining passive sensor data with active self-report measures to enhance accuracy and context sensitivity. This approach echoes recommendations by Onnela and Rauch [17], who argue that contextual data improves diagnostic precision in digital phenotyping. However, the review also highlighted gaps, including limited standardization of data collection protocols, underrepresentation of certain population groups, and the need for more robust validation of predictive models in diverse, real-world settings. This gap is also noted by Limone and Toto [12], who call for more inclusive research targeting populations with limited access to traditional mental health care. These findings suggest that future research should prioritize not only technological advancement but also methodological rigor, inclusivity, and ongoing evaluation of the clinical relevance and ethical implications of digital phenotyping.

The evaluation phase of the ESFY system provided important feedback on both usability and the broader user experience, revealing areas of strength as well as opportunities for improvement. While the majority of users were able to complete core tasks efficiently and appreciated the system’s visual design and intuitive navigation, elevated misclick and drop-off rates indicated that there remain interface elements and task flows in need of refinement, echoing the observations in [19]. These observations highlight the ongoing importance of clear guidance, user education, and iterative design, especially for first-time or less technologically fluent users. Another key outcome of the evaluation was the identification of factors shaping user trust and engagement. Users expressed a clear preference for features that foster transparency in data collection and provide opportunities for customization and control. Privacy, data accuracy, and the relationship between app-generated feedback and professional healthcare advice were recurring concerns. These concerns mirror findings from Ivanova et al. [5], who observed that users often question the reliability and ethical use of personal health data in mobile mental health apps. These findings suggest that future systems should prioritize transparent communication, robust privacy protections, and the positioning of digital health tools as complements, rather than replacements, to clinical care. User suggestions also pointed toward promising avenues for system enhancement, such as the integration of voice-based interactions, features to support digital wellness (e.g., app lock functionality), and personalized self-reflection tools like digital diaries. These features reflect a broader trend in mental health technology toward personalization and digital self-regulation, as seen in studies on mobile app addiction and usage patterns [8,9,22]. Incorporating these elements can further strengthen engagement, support self-management, and address evolving expectations for digital mental health interventions. Ultimately, a user-centered, flexible, and transparent approach to system design will be crucial for maximizing both the uptake and the impact of digital phenotyping tools.

Drawing on the insights gained from this research, several actionable recommendations and design implications emerge for the development and deployment of future digital phenotyping systems targeting psychological distress.

### 5.1. Privacy and User Control

Strong data privacy and security measures must remain a top priority, with clear, user-friendly options for managing consent and controlling the flow of personal information. To further enhance both relevance and effectiveness, digital phenotyping platforms should be designed for flexibility, allowing users to customize which data are collected, how these data are interpreted, and what types of interventions are delivered. Systems should incorporate real-time feedback and explainable AI features to help users understand how their data translates into actionable mental health insights. Such transparency not only enhances user trust but also empowers individuals to make informed decisions about their well-being. This emphasis on privacy and user control directly reflects concerns raised by participants during our evaluation of the ESFY system, where users consistently highlighted the importance of knowing what data is being collected and how it is used. These concerns echo findings from prior research that identified trust, transparency, and perceived surveillance as major barriers to adoption in mobile mental health technologies [19]. In the broader context of digital phenotyping, privacy remains a complex issue. As noted in the literature, the continuous, passive collection of behavioral data can blur the boundaries between user empowerment and perceived intrusion [16]. While such data can offer early insights into psychological distress, users must remain in control of how this information is collected, stored, and applied. This is especially critical for youth populations, who may be more vulnerable to psychological distress [7].

### 5.2. Integration with Healthcare Ecosystems

Digital phenotyping tools should be closely linked with professional healthcare services and community support networks to boost their credibility and practical value. Integrating these systems with traditional mental health care enables timely intervention, improved care coordination, and more personalized support for users, ensuring that digital solutions effectively complement, rather than replace, established care pathways and clinical expertise. This supports Insel’s argument that digital tools should enhance, not substitute, the therapeutic alliance and professional care systems [18]. Our study findings highlight this point, as users often expressed a desire for reassurance that the app’s insights were aligned with clinically validated practices and could be shared with healthcare professionals if needed. This mirrors concerns raised in previous research that digital tools, while convenient, may lack legitimacy without clear pathways to clinical integration [5]. Moreover, in the wake of the COVID-19 pandemic, where access to traditional mental health services became more limited, the need for hybrid models that bridge digital tools and professional care has become even more urgent [6,13,14]. For digital phenotyping systems like the ESFY, establishing interoperability with healthcare infrastructures and referral systems will be essential for maximizing their real-world utility and ensuring that early detection leads to effective support.

### 5.3. Personalization and Inclusivity

Finally, future system refinements should be guided by a commitment to inclusivity and personalization. This includes designing interventions that are accessible and beneficial for diverse populations, particularly those who may face greater barriers to technology adoption, such as older adults [6,12] or individuals from different cultural contexts. In addition, comprehensive onboarding and ongoing in-app guidance are essential for supporting users with varying levels of digital literacy, helping to reduce confusion and minimize attrition. Adaptive interfaces, language localization, and culturally sensitive content can all help ensure that digital phenotyping technologies address the needs of a broad user base. These considerations are particularly critical in the context of psychological distress, which affects people across all age groups and cultural backgrounds yet manifests in context-specific ways. As highlighted in the introduction, the pandemic has exacerbated mental health challenges globally [6,14], making it essential for digital tools to be both scalable and sensitive to individual needs. Our user feedback also indicated that personalization features—such as customizable self-reflection tools, adaptive feedback, and control over data interpretation—enhanced both engagement and perceived relevance. This aligns with prior research suggesting that personalized digital interventions are more effective in fostering user commitment and improving outcomes [8]. As such, inclusivity and personalization should not be treated as optional enhancements but as core design principles for effective digital phenotyping systems.

### 5.4. Limitations and Future Work

While this study offers valuable insights into the development and evaluation of digital phenotyping systems for psychological distress, several limitations must be acknowledged. The participant sample, although sufficient for initial usability evaluation, was limited in size and demographic diversity, with a predominance of female university students from a specific cultural context. This restricts the generalizability of the findings and highlights the need for future research involving broader, more heterogeneous populations. The study also relied on self-reported and simulated behavioral data within a controlled, prototype environment. As such, real-world factors such as sustained engagement over time, ecological validity of passive sensing, and integration with clinical workflows were not fully addressed. In the user study phase, sampling bias is a concern, as the participant group may not reflect the full demographic and psychological diversity of the broader youth population.

Additionally, while initial results support the feasibility and acceptability of the ESFY system, further work is required to assess its effectiveness in routine use and its ability to produce meaningful mental health outcomes outside of a research setting. Future research should aim to address these gaps through larger-scale, longitudinal studies that track real-world usage and psychological outcomes over extended periods. Clinical validation of predictive models, exploration of cross-cultural applicability, and assessment of long-term engagement and impact will be essential for translating the promise of digital phenotyping into sustainable mental health interventions. Further investigation into ethical, legal, and societal implications, particularly concerning privacy, consent, and user empowerment, remains a critical priority as the field continues to evolve.

Furthermore, the scoping review was not registered in a formal registry such as PROSPERO. While registration is not mandatory for scoping reviews, its absence may limit transparency and replicability. Future reviews will consider registration to enhance methodological rigor and adherence to open-science practices. Also, in the scoping review, selection bias may have been introduced through the inclusion criteria and a focus on peer-reviewed academic sources found only in Google Scholar, potentially overlooking relevant studies. While Google Scholar offers broad multidisciplinary coverage and includes both peer-reviewed and gray literature, this approach may have limited the comprehensiveness and reproducibility of the search. The exclusion of other academic databases such as PubMed, Scopus, and Web of Science may have resulted in the omission of relevant studies. Future reviews will incorporate multiple databases to enhance the breadth and rigor of the literature search. A key limitation in the deployment of digital phenotyping systems like the ESFY is the potential sensitivity of the data involved. Behavioral and mental health data, even when anonymized, pose significant ethical and privacy challenges. Although the current prototype is used strictly for research and evaluation purposes and does not store or transmit personal user data externally, future implementations must prioritize robust data governance frameworks. This includes clearly defining who owns the data, how it is stored, and whether it can be shared.

## 6. Conclusions

This research demonstrates the potential of digital phenotyping, grounded in both literature synthesis and user-centered design, to support the early detection and self-management of psychological distress. By systematically reviewing recent advances in digital phenotyping, translating evidence-based insights into the ESFY system, and empirically evaluating user experience and perceptions, the study provides a holistic view of both the opportunities and the challenges inherent in this rapidly evolving field. The findings highlight the value of integrating active and passive data streams, prioritizing transparency and user empowerment, and designing adaptable systems that respond to the diverse needs and concerns of end users. Moving forward, successful digital phenotyping interventions will require not only technical innovation but also careful attention to ethical, practical, and cultural considerations. The recommendations outlined in this study serve as a foundation for the continued development of effective digital mental health solutions.

## Data Availability

The original contributions presented in this study are included in the article. Further inquiries can be directed to the corresponding author.
